# Development of machine learning models aiming at knee osteoarthritis diagnosing: an MRI radiomics analysis

**DOI:** 10.1186/s13018-023-03837-y

**Published:** 2023-05-20

**Authors:** Tingrun Cui, Ruilong Liu, Yang Jing, Jun Fu, Jiying Chen

**Affiliations:** 1 Medical School of Chinese PLA, Beijing, China; 2 Department of Bone and Joint Surgery, Jining No. 2 People’s Hospital, Jining, Shandong China; 3 Department of Orthopaedics, The First Medical Centre of Chinese PLA General Hospital, Beijing, China; 4 Huiying Medical Technology Co. Ltd, Beijing, China

**Keywords:** KOA diagnosis, Magnetic resonance imaging (MRI), Machine learning, Radiomics

## Abstract

**Background:**

To develop and assess the performance of machine learning (ML) models based on magnetic resonance imaging (MRI) radiomics analysis for knee osteoarthritis (KOA) diagnosis.

**Methods:**

This retrospective study analysed 148 consecutive patients (72 with KOA and 76 without) with available MRI image data, where radiomics features in cartilage portions were extracted and then filtered. Intraclass correlation coefficient (ICC) was calculated to quantify the reproducibility of features, and a threshold of 0.8 was set. The training and validation cohorts consisted of 117 and 31 cases, respectively. Least absolute shrinkage and selection operator (LASSO) regression method was employed for feature selection. The ML classifiers were logistic regression (LR), K-nearest neighbour (KNN) and support vector machine (SVM). In each algorithm, ten models derived from all available planes of three joint compartments and their various combinations were, respectively, constructed for comparative analysis. The performance of classifiers was mainly evaluated and compared by receiver operating characteristic (ROC) analysis.

**Results:**

All models achieved satisfying performances, especially the Final model, where accuracy and area under ROC curve (AUC) of LR classifier were 0.968, 0.983 (0.957–1.000, 95% CI) in the validation cohort, and 0.940, 0.984 (0.969–0.995, 95% CI) in the training cohort, respectively.

**Conclusion:**

The MRI radiomics analysis represented promising performance in noninvasive and preoperative KOA diagnosis, especially when considering all available planes of all three compartments of knee joints.

**Supplementary Information:**

The online version contains supplementary material available at 10.1186/s13018-023-03837-y.

## Introduction

Being one of the commonest among joint diseases, knee osteoarthritis (KOA) is generally first noticed via a series of clinical manifestations (i.e. pain, tenderness, motion limitation, bone swelling, joint deformity, instability, proprioception loss, etc.) rather than with imaging manners, which most occur before symptoms do [1, 2]. De facto, all the formers do not always occur for subjects in early phases, and once were the symptoms hence atypical, the latter, generally referring to plain filming by computed radiography (CR) and magnetic resonance imaging (MRI), could be utilised to further confirm arthritis situations [3, 4].

The imaging techniques provide us opportunities to recognise early pathological changes of the affected joints. CR films display osteophytes, narrowed joint spaces and altered subchondral bone mineral density (BMD) [5, 6]. As regards MRI, comparing with CR, could better recognise more subtle pathological changes such as bone oedema, cartilage lesion and ligament injury, which are important in evaluation and classification of KOA [5–7].

Radiomics is a burgeoning batch of strategies adopting machine learning (ML) stuffs and high-flux automated extractions and analyses of interested quantitative data from clinical imaging outcomes [8, 9], and MRI radiomics is particularly more accounted of for its delicate resolution in aquiferous tissues [5–7]. However, has it been preliminarily applied in oncology in terms of diagnosis, staging and evaluation [9–12], the applications of radiomics in KOA have just gotten off the ground.

There have been a respectable number of CR radiomics studies on KOA or related issues, some of which devoted to detection and classification of KOA itself [13–17], while others provided with patterns for discovery or evaluation of related pathological changes [18, 19], exempli gratia, subchondral bone changes and cartilage loss. On the other hand, studies concerning MRI radiomics analyses on KOA, which most investigated features extracted from articular cartilage [20–23], subchondral bone [24–26] or infrapatellar fat [27–29] et al. for KOA identification, onset detection or progression evaluation, have been growing conspicuous mostly due to advantages of MRI over CR. Nevertheless, present studies gave more priority to casting in different ways on off-the-peg scoring systems determining severity or progression stages of the KOA [20, 21, 25–29] or to simply evaluating pathological changes shown in MRI images [22–24], which appeared not quite immediate or completed for diagnosis of the disease of KOA itself.

Consequently, the purpose of our study was to validate efficacy of MRI radiomics strategies in KOA evaluation, to confirm features of which combination(s) of compartments of the knee show better performance and to explore the ML models which were potentially available for practical utilities, that is, direct inference of KOA diagnoses.

## Materials and methods

### Patients

This retrospective study consecutively enrolled 148 patients with single knee MRI images acquired during the month of September, 2021. The subjects were divided into the KOA and non-KOA groups in line with the KOA diagnostic codes in Guideline (of China) for diagnosis and management of osteoarthritis (2018 edition) (Table 1) [30]. There were 78 left knees and 70 right included in total; the KOA group included 72 cases (34 males, 38 females; 39 left, 33 right; mean age, 52.32 ± 13.95 years; range, 23–83 years). The non-KOA group included 76 case (61 males, 15 females; 39 left, 37 right; mean age, 33.16 ± 11.24 years; range, 20–81 years). The data of body mass index (BMI, 24.30 ± 1.98 kg/m^2^, derived from body weight [67.85 ± 7.84 kg] and height [1.67 ± 0.09 m]), were also collected, yet those of only 53 subjects out of 148 (35.8%) were available, for these statistics are not routinely acquired at clinic of our centre.

**Table 1:** KOA diagnostic codes in Guidelines (of China) for Diagnosis and Management of Osteoarthritis (2018 edition) [30]

No	Manifestations
1	Repeated pain of knee within 1 month
2	Narrowed joint space, subchondral osteosclerosis and (or) cystic degeneration, osteophyte formation on joint margin shown in weight-bearing CR images
3	≥ 50 y/o
4	Morning stiffness ≤ 30 min
5	Bony crepitus/ friction feeling during activity

Diagnosis confirmed when suffice No.1 + (≥ 2 items among No.2, 3, 4, 5)

### Image data acquisition

All MR images were obtained with 1.5 T MR scanners (EchoStar 16-channel head coil, Alltech Medical Systems, Chengdu, China; Signa Highspeed 8-channel head coil, GE Healthcare, Milwaukee, USA). The MR protocol included fast spin-echo (FSE) T1-weighted images (T1WI) plus FSE T2-weighted images (T2WI) in the axial, coronal and sagittal planes.

### Image segmentation

**Fig. 1:**
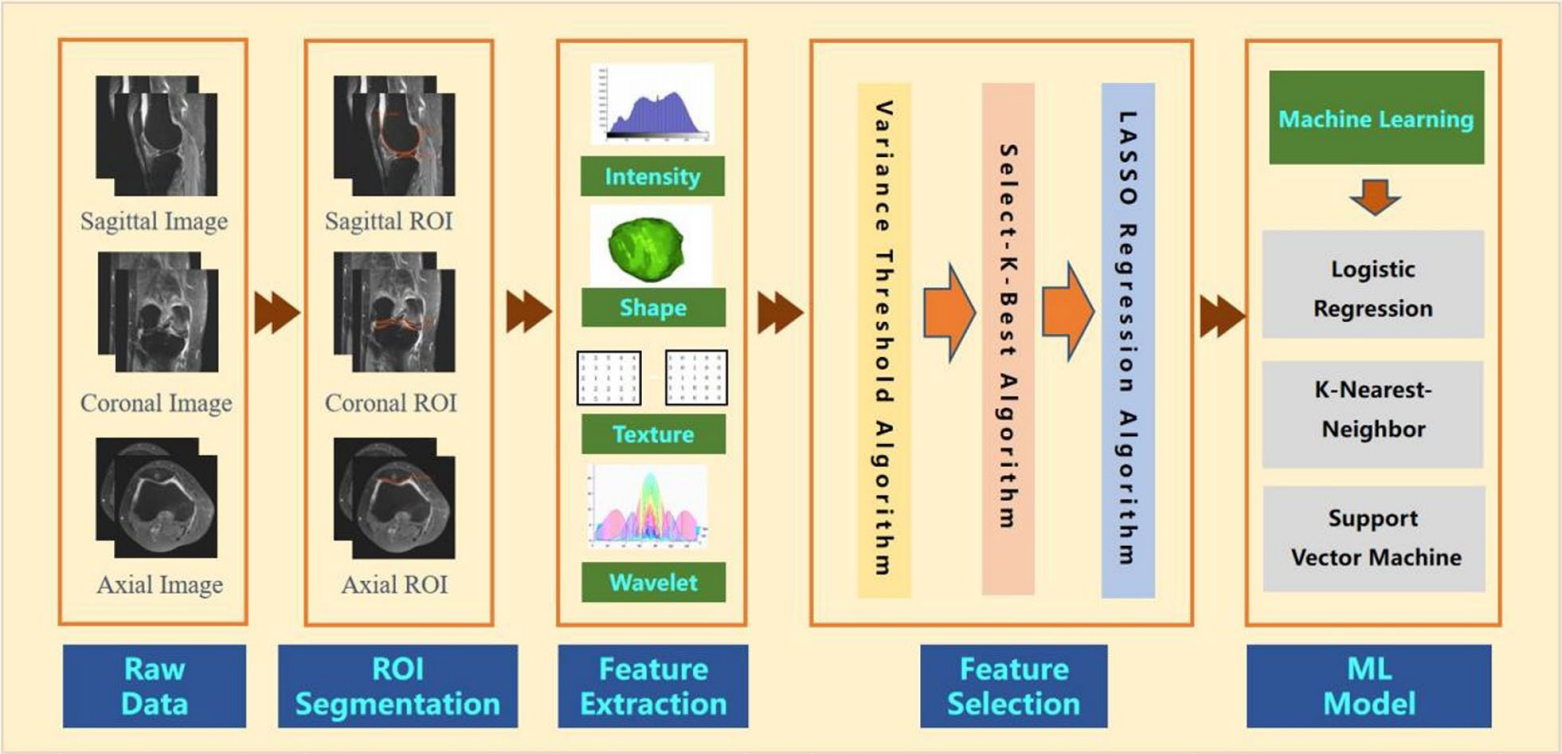
A flow-chart presenting raw-image preparation, feature extraction, feature selection and model construction

**Fig. 2:**
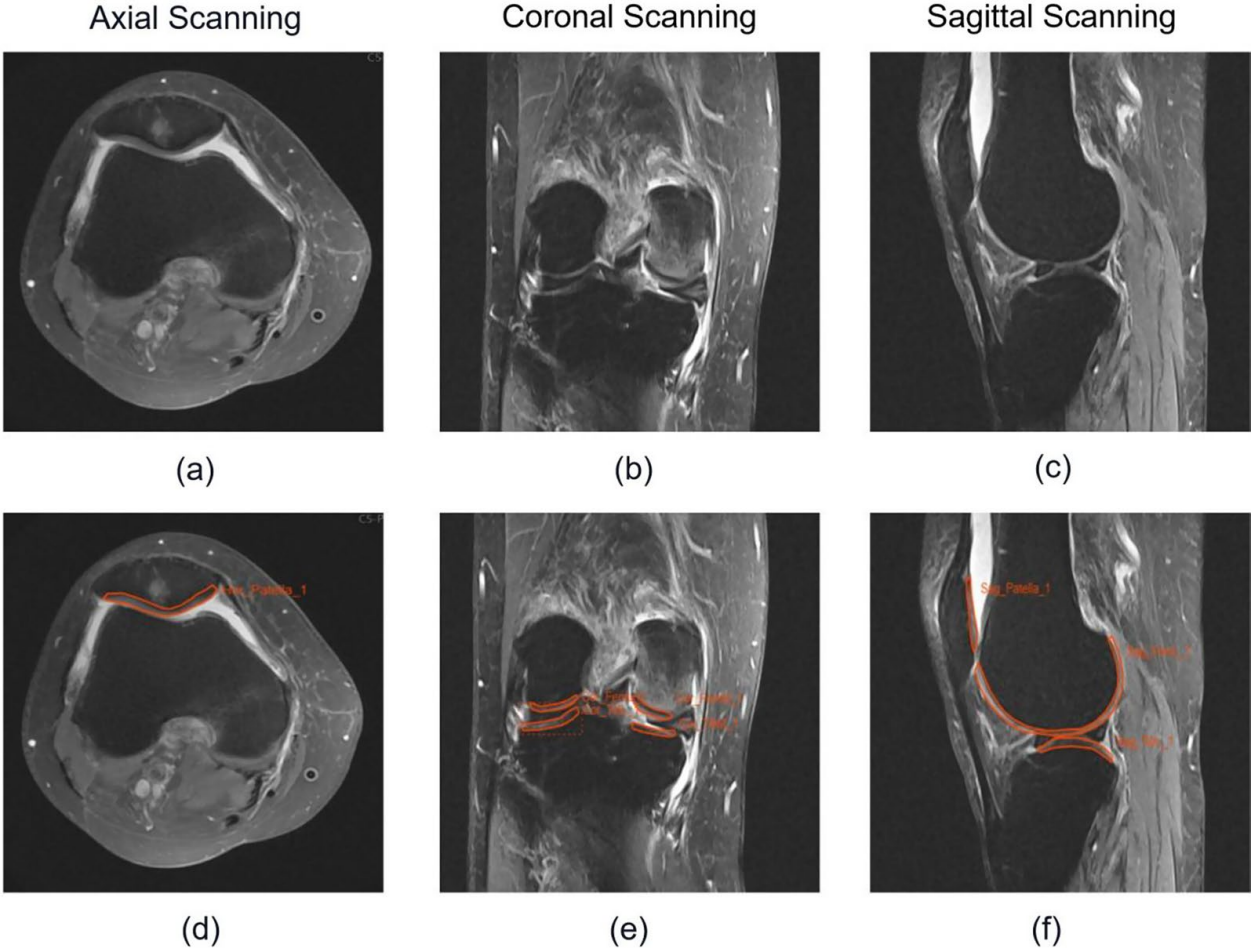
An example of manual segmentation. These were the MRI images of a female patient aged 69 y/o at clinic. Images (**a**), (**b**) and (**c**) are the original DICOM images in axial view, coronal view and sagittal view, respectively; (**d**), (**e**) and (**f**) are the manual annotation diagrams of (**a**), (**b**) and (**c**), respectively

A flow chart depicting image preparation, feature extraction, feature selection and model construction is presented in Fig. 1. To obtain the volume of interest (VOI) for further analysis, we uploaded all data to Radcloud platform (Huiying Medical Technology Co., Ltd). The VOIs of KOA were delineated manually by a radiologist with 10 years of experience in knee imaging (radiologist 1). The delineated VOIs were from cartilage of three regions, namely the medial and lateral compartments of tibiofemoral joints and patellofemoral joints, respectively. The medial and lateral VOIs corresponded to sagittal and coronal views of the tibiofemoral surfaces, and the VOIs of the patella to the sagittal and transverse positions of the patellofemoral surfaces. Regions of interest (ROIs) were thus delineated manually in the MRI for 148 patients, and VOIs were constructed by piling the slices of the corresponding ROIs in sequence. Thirty patients (with all VOIs delineated by radiologist 1) were then randomly selected from all subjects, and all VOIs were again delineated by a senior radiologist with 15 years of experience in imaging the knee joint (radiologist 2) for these patients. The interclass correlation coefficient (ICC) among 1049 features of each sequence was calculated for the latter 30 patients. ICC greater than 0.80 was considered as in good agreement, and radiomic features with ICC below 0.8, which are generally considered to be unreproducible among radiologists, were deleted [31–33]. Eventually, the work of radiologist 1 was used for further analysis. The two radiologists were blinded to the information of each subject. An example of the manual segmentation is shown in Fig. 2.

### Feature extraction

For MR image data, 1049 radiomic features were extracted from MR image data using a tool (Features Calculation) from the Radcloud platform (https://mics.huiyihuiying.com/#/subject). All the extracted radiomic features came from four categories: first-order statistical features, shape features, texture features and higher-order statistical features. First-order statistics described the intensity information of ROIs in the MR images such as maximum, median, mean, standard deviation, variance and range. Shape features reflected the shape and size of the region, such as volume, compactness, maximal diameter and surface area. Texture features could quantify regional heterogeneity differences. Higher-order statistical features consisting of the texture and intensity features produced by filtering transformation and wavelet transformation of the original MR Images: exponential, square, square root, logarithm and wavelet. Features are compliance with definitions as defined by the imaging biomarker standardisation initiative (IBSI) [34].

### Feature selection

All datasets were used to assign 80% of datasets to the training cohort and 20% of datasets to the validation cohort. Optimal features were selected from the training cohort. Prior to the steps of feature selection, all radiomic features were standardised using the StandardScaler function (in Python) by removing the mean and dividing by its standard deviation, and each set of feature value was converted to a mean of 0 with a variance of 1. Although radiomic features with ICC lower than 0.80 were removed, there still remained a great quantity of features. To improve the accuracy of model prediction and reduce the influence of features redundancy, it is necessary to remove redundant features and select the optimal features. The variance threshold method (variance threshold = 0.8) and Select-K-Best method were adopted. The Select-K-Best method used *P* < 0.05 to determine optimal features related to the KOA. The least absolute shrinkage and selection operator (LASSO) regression method was used to decrease the degree of redundancy and irrelevance. The optimal $\alpha$, which is the coefficient of regularisation in the LASSO method, was selected using inner tenfold cross-validation in the training cohort with the maximum iteration of 5000 via minimum average mean square error (MSE). Subsequently, the radiomics parameters with nonzero coefficients in the LASSO algorithm generated by the whole training cohort with the optimal $\alpha$ were selected.

### Model construction

The selected features were taken as the inputs for model construction to differentiate KOA from all patients. Images were classified as KOA or non-KOA using ML methods in combination with the selected features listed above. Models were constructed with ML algorithms including logistic regression (LR), K-nearest neighbour (KNN) and support vector machine (SVM) in the training cohort. In the process of model building, every classifier was tuned and the hyperparameters were optimised to maximise the diagnostic performance. In SVM algorithm, the hyperparameters of C (including 0.1, 0.8, 0.5, 1, 3, 5) and kernel (‘rbf’, ‘linear’, ‘sigmoid’) were included; in KNN algorithm, they were n_neighbours (the range is from 2 to 10) and algorithm (‘auto’, ‘ball_tree’, ‘kd_tree’); and in LR algorithm, the included hyperparameters were penalty (‘l1’, ‘l2’) and C (including 0.1, 0.5, 0.8, 1, 3, 5). The classification results were evaluated with a receiver operating characteristic (ROC) curve with the associated area under the ROC curve (AUC), accuracy, sensitivity and specificity.

In a single algorithm, 11 models were, respectively, constructed for comparative analysis. Three models of medial tibiofemoral VOIs were constructed, respectively, including sagittal model (M-S model), coronal model (M-C model) and combined model of the sagittal-coronal (M-S-C model). Similarly, three models of lateral tibiofemoral VOIs were constructed, respectively, as sagittal model (L-S model), coronal model (L-C model) and combined model of the sagittal-coronal (L-S-C model). In patellar VOIs, sagittal model (P-S model), transverse model (P–T model) and combined model of the sagittal-transverse (P-S-T model) were constructed. In addition, we combined all the features to build a comprehensive model (Final model, Final-M). After training, estimations of the generalisation performance of each model were validated in the validation cohort. Besides, clinical data of age, gender and BMI were taken into the construction of an additional model for clinical statistics analyses (Clnc model) rather than being mixed into the former 10 models mainly because of obvious missing of relevant BMI statistics.

### Statistical analysis

All statistical analyses were performed using R software version 3.3.0. Normalisation of features, selection of features and model construction were undertaken using Python 3.7.0, Scikit-learn package 0.19.2 and Pyradiomics package 2.2.0. Other statistical analyses were performed using R software version 3.3.0. ROC curve analysis was used to evaluate the diagnostic performances of ML classifiers [95% confidence intervals (CIs), specificity and sensitivity were also calculated], and four indicators including P (precision = true positives/(true positives + false positives)), R (recall = true positives/(true positives + false negatives)), f1-score (f1-score = P*R*2/(P + R)), support (total number in test set) to evaluate the performance of classifier in this study. The statistical analysis was performed in Radcloud platform (https://mics.huiyihuiying.com/).

## Results

### Feature extraction and feature selection

For the M-S model, 518 features were first screened from 2098 features using the ICC test. Then, the 518 features were screened by the variance threshold algorithm (variance threshold = 0.8), Select-K-Best algorithm and Lasso algorithm, respectively. Finally, 16 optimal features were screened. By repeating the above steps, the M-C, M-S-C, L-S, L-C, L-S-C, P-S, P–T, P-S-T and Final model retained 13, 16, 21,19, 35, 28, 15, 43 and 42 features as the optimal feature set, respectively (Table 2). In the four combined models (M-S-C, L-S-C, P-S-T and Final-M), the process of LASSO algorithms is shown in Additional file 1: Fig. 1.

**Table 2:** The process of feature selection

	M-S	M-C	M-S-C	L-S	L-C	L-S-C	P-S	P–T	P-S-T	Final
Total features	2098	2098	4196	2098	2098	4196	1049	1049	2098	10,490
ICC	518	456	974	551	349	900	558	730	1288	3162
LASSO	16	13	16	21	19	35	28	15	43	42
Optimal feature set	16	13	16	21	19	35	28	15	43	42

### Performance of the diagnosis models in predicting the KOA

The results of KNN algorithm, LR algorithm and SVM algorithm are shown in Table 3. In general, all the models achieved satisfying performance, especially in the combined model (Final model), where accuracy and AUC of LR classifier were 0.968, 0.983 (0.957–1.000, 95% CI) in the validation cohort, compared to 0.940 and 0.984 (0.969–0.995, 95% CI) in the training cohort, respectively.

**Table 3:** Results of algorithms of KNN, LR and SVM

Algorithm	Model	Cohort	AUC (95% CI)	Accuracy	Sensitivity	Specificity
*KNN*	M-S	Train	0.786 (0.710–0.844)	0.701	0.614	0.783
		Validation	0.712 (0.529–0.833)	0.613	0.533	0.688
	M-C	Train	0.805 (0.741–0.863)	0.718	0.544	0.883
		Validation	0.771 (0.621–0.886)	0.645	0.533	0.750
	M-S-C	Train	0.866 (0.809–0.915)	0.786	0.667	0.900
		Validation	0.860 (0.724–0.952)	0.839	0.733	0.938
	L-S	Train	0.832 (0.773–0.888)	0.744	0.667	0.817
		Validation	0.706 (0.530–0.83)	0.677	0.533	0.875
	L-C	Train	0.835 (0.770–0.886)	0.735	0.649	0.817
		Validation	0.752 (0.592–0.895)	0.710	0.667	0.750
	L-S-C	Train	0.867 (0.890–0.912)	0.778	0.632	0.917
		Validation	0.796 (0.625–0.931)	0.839	0.733	0.938
	P-S	Train	0.774 (0.707–0.844)	0.667	0.561	0.767
		Validation	0.694 (0.559–0.867)	0.677	0.553	0.813
	P–T	Train	0.834 (0.762–0.889)	0.769	0.702	0.833
		Validation	0.721 (0.598–0.891)	0.677	0.600	0.750
	P-S-T	Train	0.846 (0.786–0.86)	0.769	0.684	0.850
		Validation	0.950 (0.890–0.993)	0.903	0.933	0.875
	Final-M	Train	0.927 (0.878–0.960)	0.880	0.789	0.967
		Validation	0.938 (0.862–0.988)	0.839	0.800	0.875
	Clnc-M	Train	0.695 (0.622–0.762)	0.684	0.632	0.733
		Validation	0.692 (0.531–0.827)	0.642	0.600	0.688
*LR*	M-S	Train	0.813 (0.736–0.872)	0.718	0.737	0.700
		Validation	0.883 (0.745–0.996)	0.742	0.733	0.750
	M-C	Train	0.774 (0.696–0.840)	0.726	0.684	0.767
		Validation	0.733 (0.567–0.885)	0.710	0.800	0.625
	M-S-C	Train	0.830 (0.759–0.885)	0.744	0.719	0.767
		Validation	0.875 (0.754–0.962)	0.774	0.733	0.813
	L-S	Train	0.876 (0.819–0.924)	0.795	0.789	0.800
		Validation	0.913 (0.804–0.983)	0.806	0.733	0.875
	L-C	Train	0.839 (0.771–0.895)	0.761	0.702	0.817
		Validation	0.842 (0.704–0.950)	0.742	0.800	0.688
	L-S-C	Train	0.917 (0.873–0.952)	0.821	0.789	0.850
		Validation	0.938 (0.857–0.991)	0.871	0.800	0.938
	P-S	Train	0.884 (0.829–0.931)	0.786	0.754	0.817
		Validation	0.883 (0.858–0.992)	0.806	0.867	0.750
	P–T	Train	0.885 (0.832–0.933)	0.821	0.754	0.883
		Validation	0.908 (0.836–0.982)	0.742	0.667	0.813
	P-S-T	Train	0.977 (0.957–0.993)	0.932	0.947	0.917
		Validation	0.921 (0.906–1.000)	0.806	0.867	0.750
	Final-M	Train	0.984 (0.969–0.995)	0.940	0.877	1.000
		Validation	0.983 (0.957–1.000)	0.968	1.000	0.938
	Clnc-M	Train	0.684 (0.599–0.751)	0.684	0.544	0.817
		Validation	0.644 (0.451–9.782)	0.645	0.533	0.451
*SVM*	M-S	Train	0.829 (0.752–0.885)	0.752	0.737	0.767
		Validation	0.708 (0.521–0.850)	0.645	0.600	0.689
	M-C	Train	0.883 (0.826–0.929)	0.821	0.772	0.867
		Validation	0.792 (0.647–0.919)	0.742	0.800	0.689
	M-S-C	Train	0.885 (0.822–0.931)	0.769	0.737	0.800
		Validation	0.817 (0.649–0.929)	0.710	0.733	0.688
	L-S	Train	0.920 (0.881–0.953)	0.821	0.789	0.850
		Validation	0.838 (0.693–0.940)	0.774	0.667	0.875
	L-C	Train	0.888 (0.833–0.930)	0.786	0.719	0.850
		Validation	0.829 (0.675–0.947)	0.806	0.800	0.813
	L-S-C	Train	0.941 (0.905–0.970)	0.821	0.754	0.883
		Validation	0.896 (0.765–1.000)	0.839	0.800	0.875
	P-S	Train	0.923 (0.883–0.959)	0.821	0.772	0.867
		Validation	0.867 (0.832–0.986)	0.806	0.733	0.875
	P–T	Train	0.915 (0.864–0.953)	0.838	0.789	0.883
		Validation	0.858 (0.744–0.975)	0.806	0.733	0.875
	P-S-T	Train	0.956 (0.927–0.978)	0.846	0.789	0.900
		Validation	0.879 (0.827–1.000)	0.806	0.733	0.875
	Final-M	Train	0.984 (0.968–0.996)	0.940	0.877	1.000
		Validation	0.958 (0.895–1.000)	0.935	0.933	0.938
	Clnc-M	Train	0.747 (0.674–0.815)	0.667	0.667	0.667
		Validation	0.715 (0.548–0.860)	0.710	0.733	0.688

**Fig. 3:**
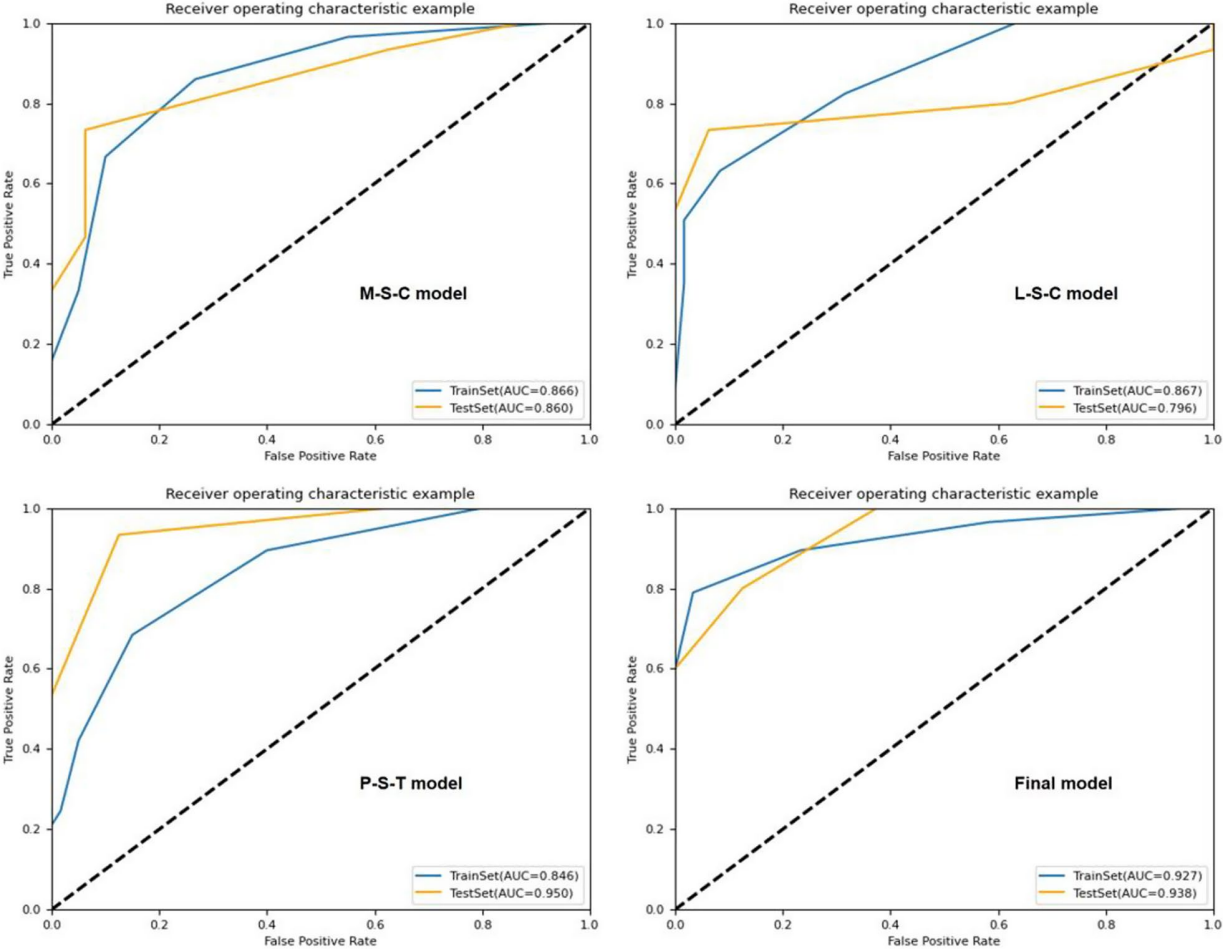
The ROC curve of KNN algorithm

**Fig. 4:**
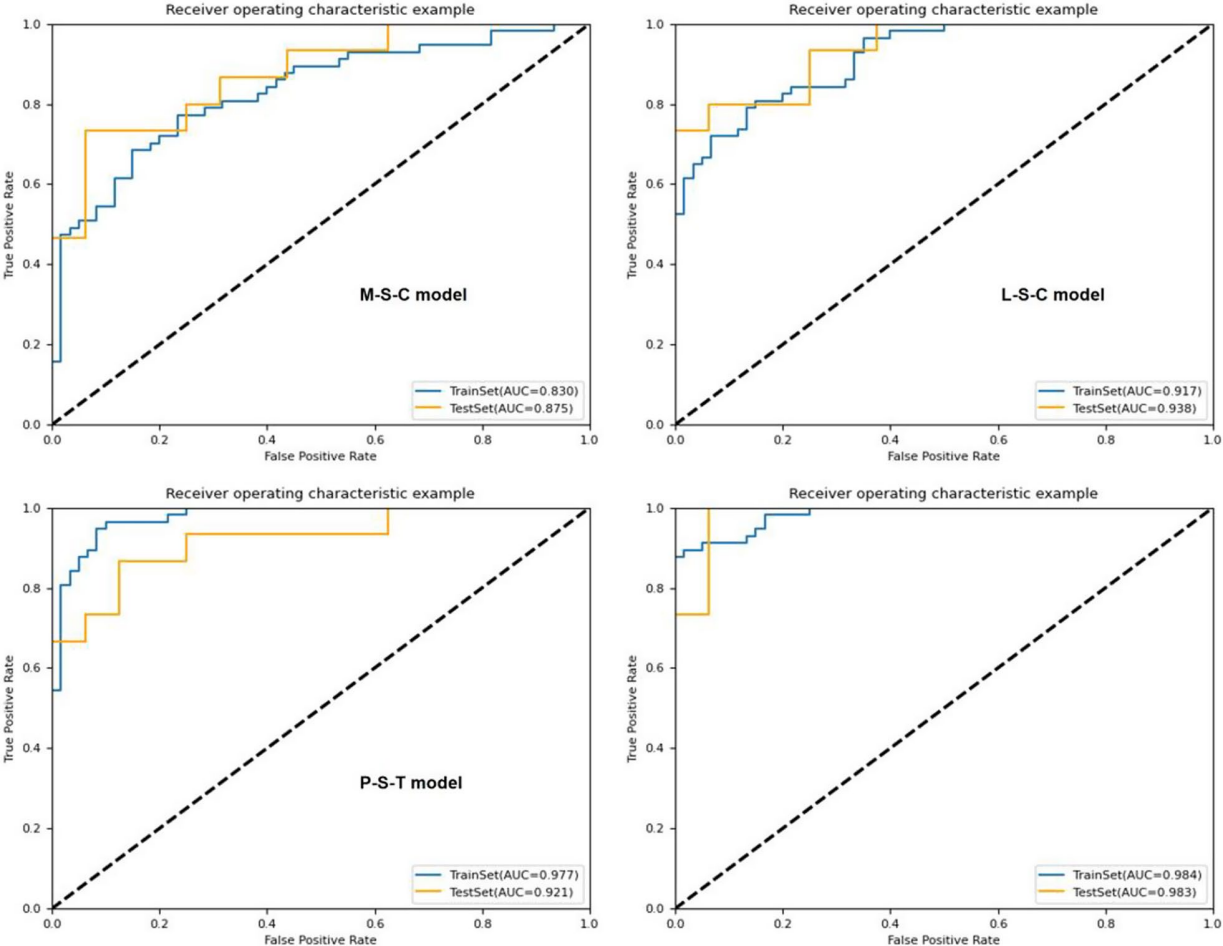
The ROC curve of LR algorithm

**Fig. 5:**
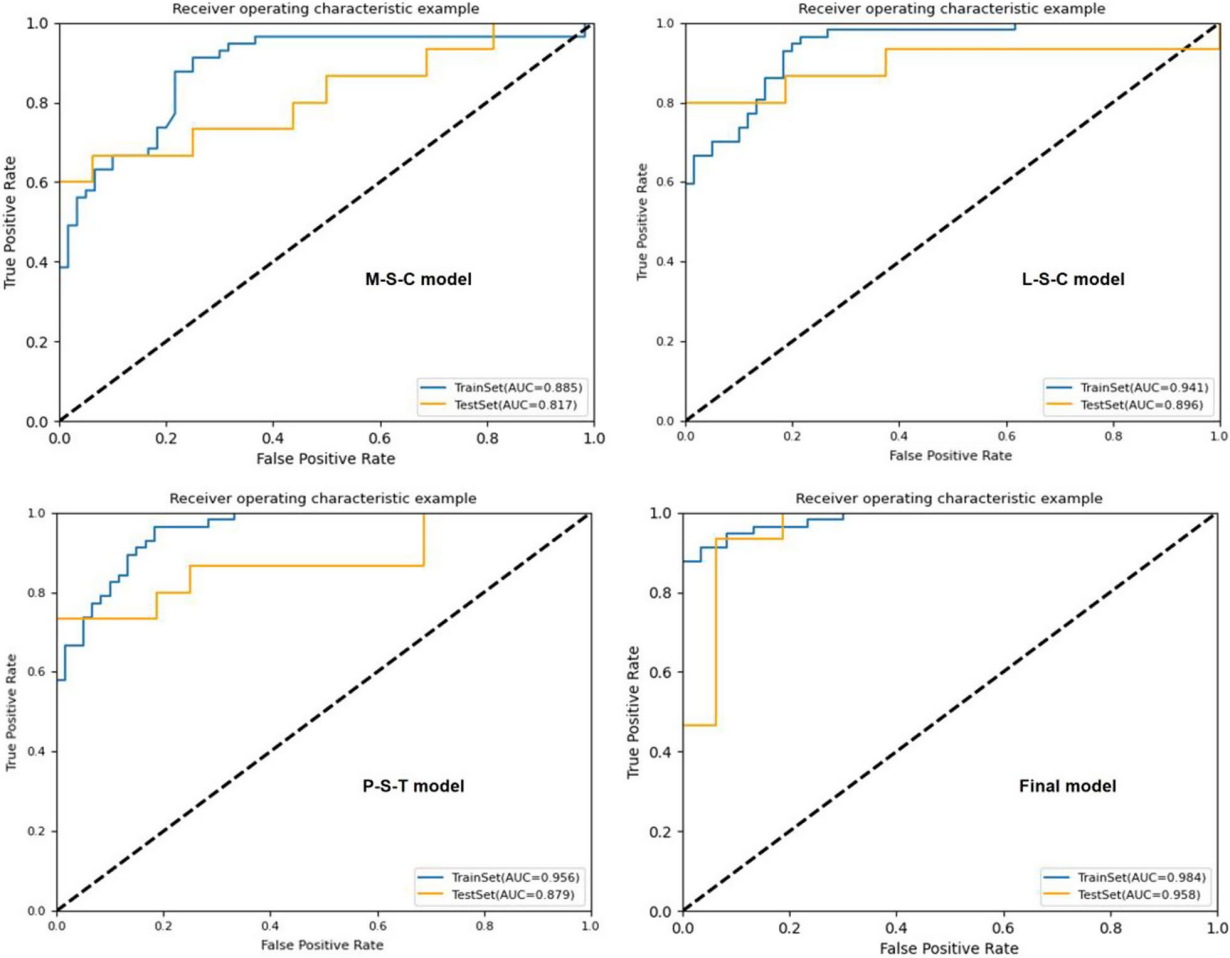
The ROC curve of SVM algorithm

**Fig. 6:**
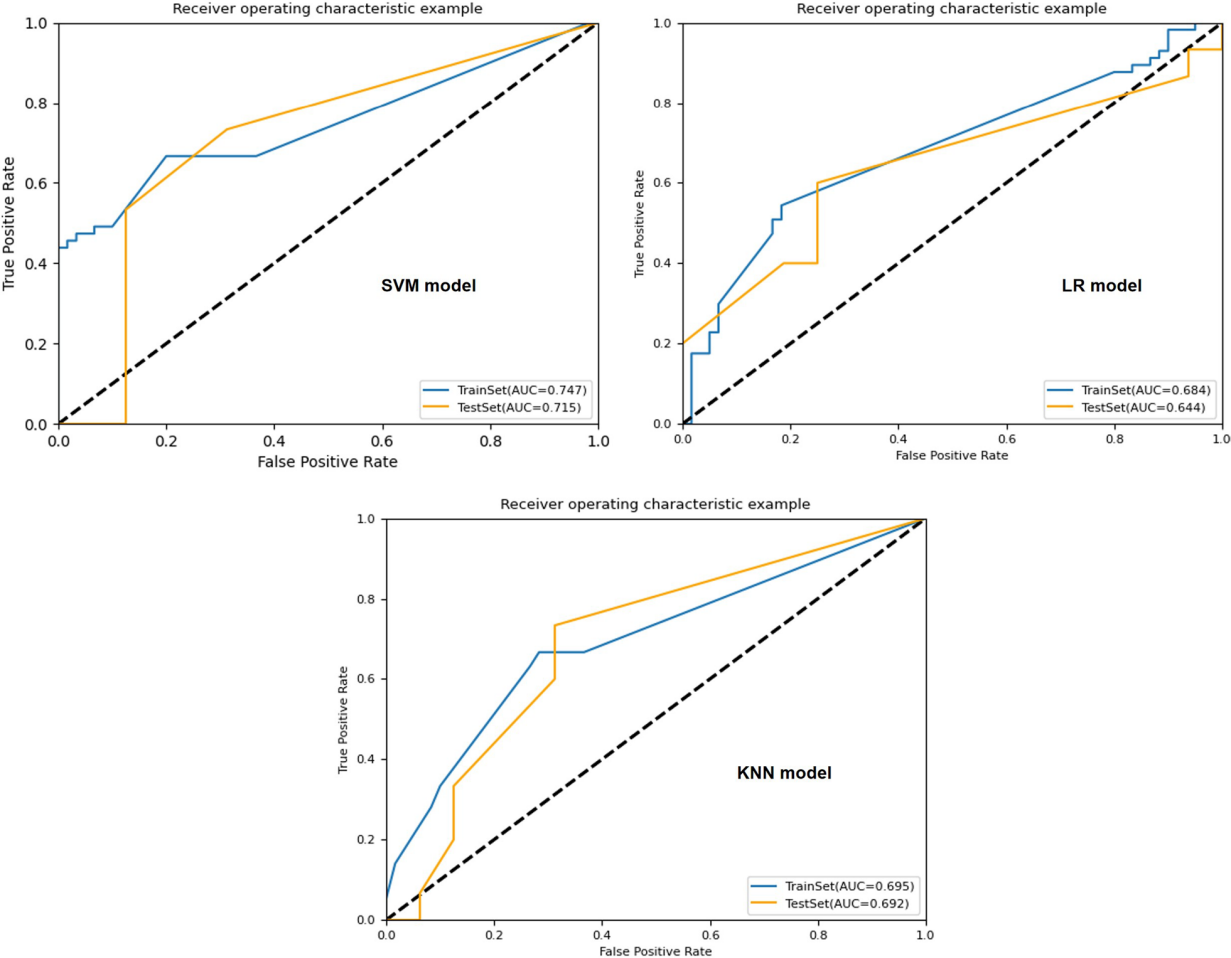
The ROC curve of the Clnc model

Among the four combined models (M-S-C, L-S-C, P-S-T and Final-M), the LR algorithm showed better performance in KOA diagnosis. In validation sets of each model, the AUC of LR algorithm ranged from 0.875 to 0.983, and the accuracy ranged from 0.774 to 0.968. The ROC curves of the four models are shown in Fig. 3, Figs. 4 and 5. However, the performance of Clnc model was apparently inferior to radiomics-based models. The SVM algorithm showed relatively more optimal performance in Clnc model, with the AUC of 0.747 in the training cohort and 0.715 in the validation cohort, respectively. The ROC curves of the Clnc model are shown in Fig. 6.

## Discussion

Our models achieved direct inferences from cartilage lesions to KOA diagnosis by enumerating and analysing filtered features extracted from MRI images of cartilage with the aid of various types of algorithms, before which the VOIs were manually, rather than automatedly, delineated by salted radiologists. There exist studies on automated ROI/VOI selection and evaluation manners of MRI images in KOA patients by virtue of ML. Nunes et al. [22] completed their works on automated detection and staging of cartilage lesions and bone marrow oedema, yet only included diagnosed KOA subjects. Pedoia et al. [23] developed their classification system merely on meniscal lesion. Therefore, what we have excavated in our study is hitherto relatively rare.

Radiomics studies focusing on KOA were less performed on MRI images comparing with CR. A respectable bunch of investigations based on CR image data were performed to extract meaningful features or give immediate Kellgren–Lawrence classification data, employing different algorithms in various stages [13–15], and efforts for portable devices were also set on track [35]. In clinical practice, CR is the much more used methods than MRI to screen KOA because of its convenience, economy and radiologic safety (comparing with CT of course), while MRI scanning is used in relatively rare situations to explore details of the joints and exhibit cartilage, which could hardly be shown by the former [2, 6, 36]. However, this would surely affect the continuum of the included subjects in our study, for few subjects from clinics accept MRI scans. Furthermore, despite the advantages of MRI over CR on early stage detection of pathological changes [4, 7], the sensitivity in KOA diagnosis of 61% [3] is still low, requiring standard algorithms to further solidify diagnostic effectiveness. In this regard, our study had a meaningful attempt.

To our limited knowledge, our models were innovative to some extent, in which KOA diagnoses were developed without adopting any intact ready-made scoring system. There exist several semi-quantitative scoring systems in KOA, such as Whole-Organ MRI Score (WORMS) [37] or MRI osteoarthritis knee score (MOAKS) [38], utilising artificially accessible MRI features signs of the knee. These systems were developed to manage higher effectiveness on diagnosis, and had been used as core idea in some of the radiomics studies [21–23, 26]. The crux of the matter is that any of the scoring systems were designed merely for precise diagnosis of KOA by quantifying and weighing data that could be conveniently manually acquired. Inasmuch as ML models could recognise necessary features and perform reliable analysis so that to best achieve the discrimination of the disease and even approach gold standard, we might not require a scoring system by rote anymore.

Additionally, it was concluded in our study that the more planes and compartments were picked among various permutation and combination for combined analyses, the better performances the models could achieve. The knee joints were divided into three compartments in our study, that is, lateral and medial tibiofemoral compartments as well as the patellofemoral space. An MRI radiomics study working on subchondral trabeculae developed their KOA severity assessment from four individual ROIs out of two tibiofemoral compartments of knee joints [25]. Besides diagnosis deduction issues, it is pellucid that a sole plane/ROI out of a 3-dimentional system is apt to omit necessary details, and the exact compartment(s) where the pathological changes of cartilage occur varies from patients and knees [2]. Therefore, full-scale data management would be necessary for future debugging and application of KOA radiomics diagnosis models, in the interest of both comprehensiveness of evaluation and deep going analyses of subjects with each kind of affected compartments.

Nevertheless, as any ML derived models, ours might have several ‘birth defects’ [16]. For instance, a large dataset would benefit model training [39]. The feature recognition model derived by Nunes et al. [22] brought into 1435 knees; the automated staging device developed by Suresha et al. [17] used 7549 CR images for ML progressions, and a similar model by Tiulpin et al. [14] subsumed 5960 knees. Our subject pool of 148 knees in our study was an obvious shortcoming for an ML model. Concurrently, derivation processes of the ML-based models require external validations [40]. Internal validations are essential for ML model development [22, 23], yet could not replace external validations; the latter demanding open-source software or data resources and accordingly remaining rare, would be required to help avoid selection bias [41]. Moreover, the ‘black box’ nature of ML models conceals inner logics of inference, resulting in poor understanding of the generation of any judgements [42].

In terms of radiomics strategies applied, tenfold cross-validation was used in our analyses to screen the optimal features of the radiomics features. Yet in the subsequent model construction, due to the excessive training time and calculation consumption caused by the large count of established models (which was up to 33), randomisation (in grouping), which had been utilised by former studies [12, 43], was consequently also adopted for model construction instead of cross-validation. Additionally, as a set of models aiming at serving rapid, automatic and precise clinical diagnosis of KOA, fundamental statistics of patients, which were age, gender, BMI, etc., which ought to be included in case of good performance [44], were reluctantly discarded in general MRI radiomics analyses due to critical missing caused by the yet-to-be-standardised clinic workflow. Such loss may result in yielding in further optimisation of the radiomics models. Therefore, we are planning in future studies for data collection on a larger and all-round scale, and utility of cross-validation during grouping courses as well.

Besides the mentioned ones, numbers of limitations in our study still exist. First, such is in nature a cross-sectional study, which included no prospective contents, nor any prognosis datum. Second, because the enrolled images were directly extracted from the Digital Imaging and Communications in Medicine (DICOM) system by scanning date, the consecutiveness of subjects would be harmed and thus increased the risk of bias. Third, we simply brought features of joint cartilage condition into diagnosis derivation process, which may lead to deviation in KOA recognition due to the lack of overall estimation of joint condition.

## Conclusion

ML models for KOA diagnosis based on MRI radiomics analysis were formed via various programs and algorithms, before which the ROIs-VOIs were manually delineated. The model reached sound effects, and when combining all available planes of all three compartments of the knee joints (Final-M) and utilising the LR algorithm, AUC, accuracy, sensitivity and specificity were, respectively, achieved to be 0.984 (0.969–0.995, 95% CI), 0.940, 0.877 and 1.000 in the training cohort, and 0.983 (0.957–1.000, 95% CI), 0.968, 1.000 and 0.938 in the validation cohort, which came up to be quite satisfying, and the best outcome among training and validation consequences, respectively.

## Supplementary Information

**Additional file 1: Fig. S1.** Lasso algorithm on features select.

## Abbreviations

ML: Machine learning
MRI: Magnetic resonance imaging
KOA: Knee osteoarthritis
BMI: Body mass index
ICC: Intraclass correlation coefficient
LASSO: Least absolute shrinkage and selection operator
LR: Logistic regression
KNN: K-nearest neighbour
SVM: Support vector machine
ROC: Receiver operating characteristic
AUC: Area under curve
CI: Confidence interval
CR: Computed radiography
BMD: Bone mineral density
VOI: Volume of interest
ROI: Regions of interest
ICC: Interclass correlation coefficient
IBSI: Imaging biomarker standardisation initiative
LASSO: Least absolute shrinkage and selection operator
LR: Logistic regression
KNN: K-nearest neighbour
SVM: Support vector machine
WORMS: Whole-organ MRI score
MOAKS: MRI osteoarthritis knee score
DICOM: Digital imaging and communications in medicine
