# Supplementary figures and images for: Development of machine learning models aiming at knee osteoarthritis diagnosing: an MRI radiomics analysis
Source: J Orthop Surg Res. 2023 May 20;18:375. doi: 10.1186/s13018-023-03837-y (PMC10199595; doi:10.1186/s13018-023-03837-y)

Supplement Fig.1. Lasso algorithm on features select.


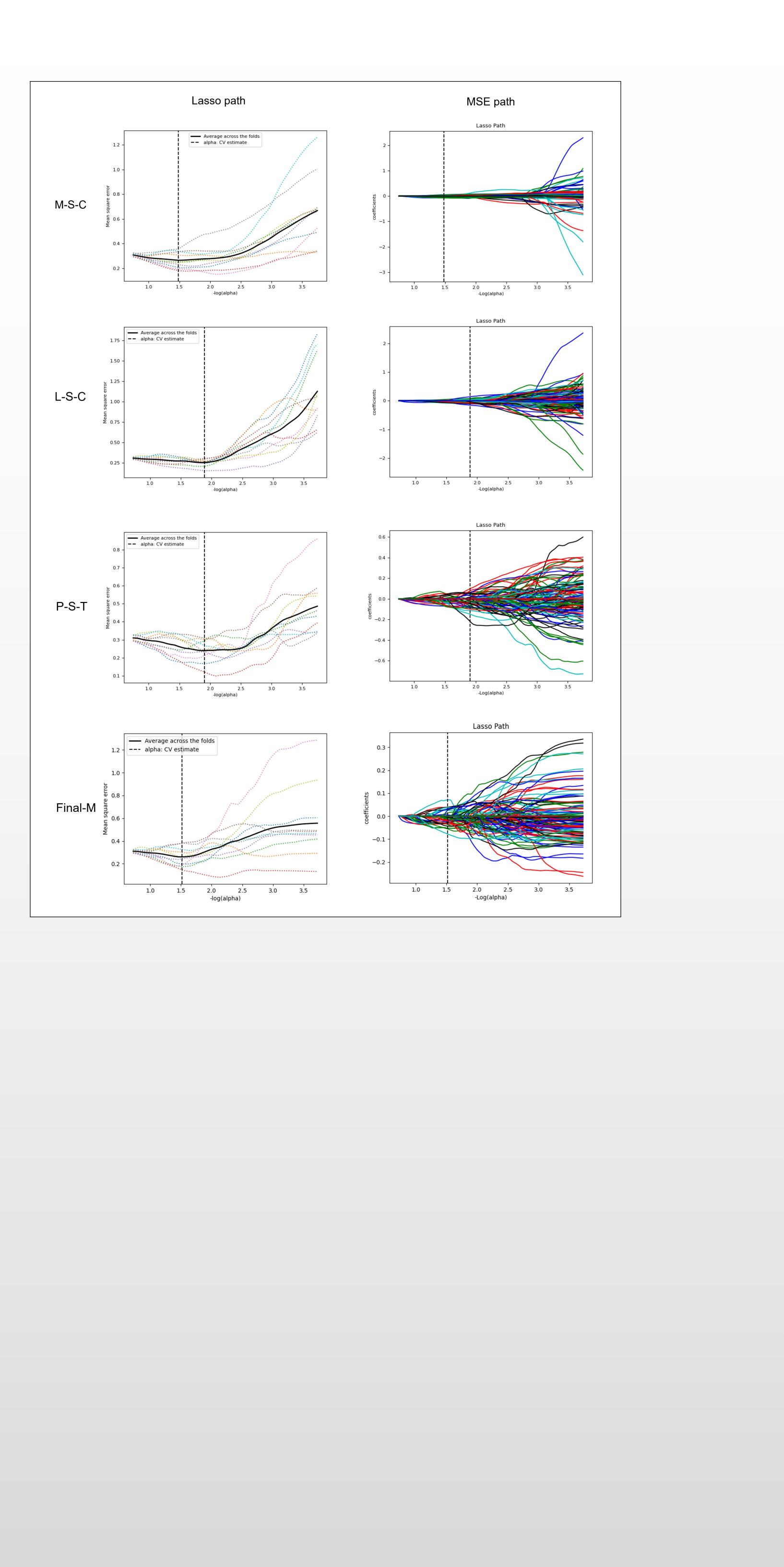

Supplement: Supplementary file 1 — Additional file 1: Fig. S1. Lasso algorithm on features select. [file 13018_2023_3837_MOESM1_ESM.docx]
